# Artificial Intelligence-Assisted Biparametric MRI for Detecting Prostate Cancer—A Comparative Multireader Multicase Accuracy Study

**DOI:** 10.3390/jcm14176111

**Published:** 2025-08-29

**Authors:** Daniel Nißler, Sabrina Reimers-Kipping, Maja Ingwersen, Frank Berger, Felix Niekrenz, Bernhard Theis, Fabian Hielscher, Philipp Franken, Nikolaus Gaßler, Marc-Oliver Grimm, Ulf Teichgräber, Tobias Franiel

**Affiliations:** 1 Institute of Diagnostic and Interventional Radiology, Friedrich-Schiller-University Jena, Jena University Hospital, 07747 Jena, Germany; daniel.nissler@med.uni-jena.de (D.N.); maja.ingwersen@gmx.de (M.I.); felix.niekrenz@med.uni-jena.de (F.N.); ulf.teichgraeber@med.uni-jena.de (U.T.); 2 FUSE-AI GmbH, 20457 Hamburg, Germany; sabrina.reimers-kipping@fuse-ai.de (S.R.-K.); fabian.hielscher@fuse-ai.de (F.H.); 3 Department of Urology, Friedrich-Schiller-University Jena, Jena University Hospital, 07747 Jena, Germany; frank.berger@uk-halle.de (F.B.); marc-oliver.grimm@med.uni-jena (M.-O.G.); 4 Department of Urology, Martin-Luther-University Halle-Wittenberg, Halle (Saale) University Hospital, 06108 Halle (Saale), Germany; 5 Department of Pathology, Friedrich-Schiller-University Jena, Jena University Hospital, 07747 Jena, Germany; bernhard.theis@med.uni-jena.de (B.T.); nikolaus.gassler@med.uni-jena.de (N.G.); 6 Center for Clinical Studies, Friedrich-Schiller-University Jena, Jena University Hospital, 07747 Jena, Germany; philipp.franken@med.uni-jena.de

**Keywords:** artificial intelligence, biparametric MRI, deep learning, computer-assisted diagnosis, multiparametric magnetic resonance imaging, prostate cancer

## Abstract

**Objectives:** To evaluate the diagnostic accuracy of AI-assisted biparametric MRI (AI-bpMRI) in detecting prostate cancer (PCa) as a possible replacement for multiparametric MRI (mpMRI) depending on readers’ experience. **Methods:** This fully crossed, multireader multicase, single-centre, consecutive study retrospectively included men with suspected PCa. Three radiologists with different levels of experience independently scored each participant’s biparametric (bp) MRI, mpMRI, and AI-bpMRI according to the PI-RADS V2.1 classification. The AI-assisted image processing was based on a sequential deep learning network. Histopathological findings were used as a reference. The study evaluated the mean areas under the receiver operating characteristic curves (AUCs) using the jackknife method for covariance. AUCs were tested for non-inferiority of AI-bpMRI to mpMRI (non-inferiority margin: −0.05). **Results:** A total of 105 men (mean age 66 ± 7 years) were evaluated. AI-bpMRI was non-inferior to mpMRI in detecting both Gleason score (GS) ≥ 3 + 4 PCa (AUC difference: 0.03 [95% CI: −0.03, 0.08], *p* = 0.37) and GS ≥ 3 + 3 PCa (AUC difference: 0.04 [95% CI: −0.01, 0.09], *p* = 0.14) and was superior to bpMRI in detecting GS ≥ 3 + 3 PCa (AUC difference: 0.07 [95% CI: 0.02, 0.12], *p* = 0.004). The benefit of AI-bpMRI was greatest for the readers with low or medium experience (AUC difference in detecting GS ≥ 3 + 4 compared to mpMRI: 0.06 [95% CI: −0.03, 0.14], *p* = 0.19 and 0.06 [95% CI: −0.03, 0.14], *p* = 0.19, respectively). **Conclusions:** This study indicates that AI-bpMRI detects PCa with a diagnostic accuracy comparable to that of mpMRI.

## 1. Introduction

Prostate cancer (PCa) is a major health concern in men, with incidence and death rates of 115 and 19 per 100,000 men, respectively. This makes PCa one of the most commonly diagnosed cancers in men [1]. Early detection when the disease is still localized improves survival rates.

MRI of the prostate is strongly recommended by current guidelines prior to biopsy [2,3,4]. The most established approach is multiparametric MRI (mpMRI) [5], including both morphological (T2-weighted [T2w] imaging) and functional sequences (diffusion-weighted imaging [DWI] and dynamic contrast-enhanced imaging [DCE]). It was shown that the concordance rates regarding Gleason Score between biopsy and the radical prostatectomy histologic report and the detection of clinically significant PCa at mpMRI can achieve good and improvable accuracy [6]. Whether biparametric MRI (bpMRI), which does not include dynamic contrast-enhanced images, also provides sufficient information, even in the presence of artifacts or an inadequate signal-to-noise ratio, is still under debate [4,5]. In any case, compared to mpMRI, it saves time and costs [5] and avoids potential side effects of contrast agents [4,7]. However, for the time being, the Prostate Imaging Reporting and Data System (PI-RADS) Steering Committee recommends that mpMRI should be preferred to bpMRI when it is critical not to miss a clinically significant PCa [4]. However, it has been shown that radiologist experience is a dominant factor influencing the accuracy of mpMRI [8].

In general, AI-assisted image interpretation can address the rising demand for medical imaging worldwide [9]. Specifically, it can improve MRI by reducing scan times, minimizing variance in interpretations between radiologists, automating the out-lining of the prostate gland, and identifying intraprostatic targets before biopsy. It can also enable diagnostic predictions of probable histology [10]. In addition, research into simplification of the diagnostic sequences used in MRI, in conjunction with initial AI-based interpretation, can increase throughput, reduce reading time and costs, and improve consistency [10,11].

Recent research has demonstrated that artificial intelligence (AI) algorithms can provide reliable decision support for the PCa diagnosis at bp- and mpMRI [9,12,13,14]. If these demonstrate superior performance to radiologists, they will also gain a high level of patient acceptance [15].

However, whether and in what cases such detection AI-assisted biparametric MRI (AI-bpMRI) can replace mpMRI and obviate the need for contrast enhancement remains to be investigated. The objective of our study was to compare the diagnostic accuracy of AI-bpMRI, mpMRI, and bpMRI under consideration of the reader’s experience. The second objective was to investigate whether the accuracy of AI-bpMRI is non-inferior to that of mpMRI.

## 2. Materials and Methods

The study was approved by the institutional review committee of the Friedrich-Schiller-university *Jena* (*Jena*, Germany) before the start of the study. Written informed consent was waived due to the retrospective nature of the study and pseudonymization of participants’ data. For research purposes, the data were accessed on 30 September 2021. *Daniel Nießler* and Tobias Franiel had access to information that could identify individual participants after data collection.

### 2.1. Study Cohort

**Figure 1:**
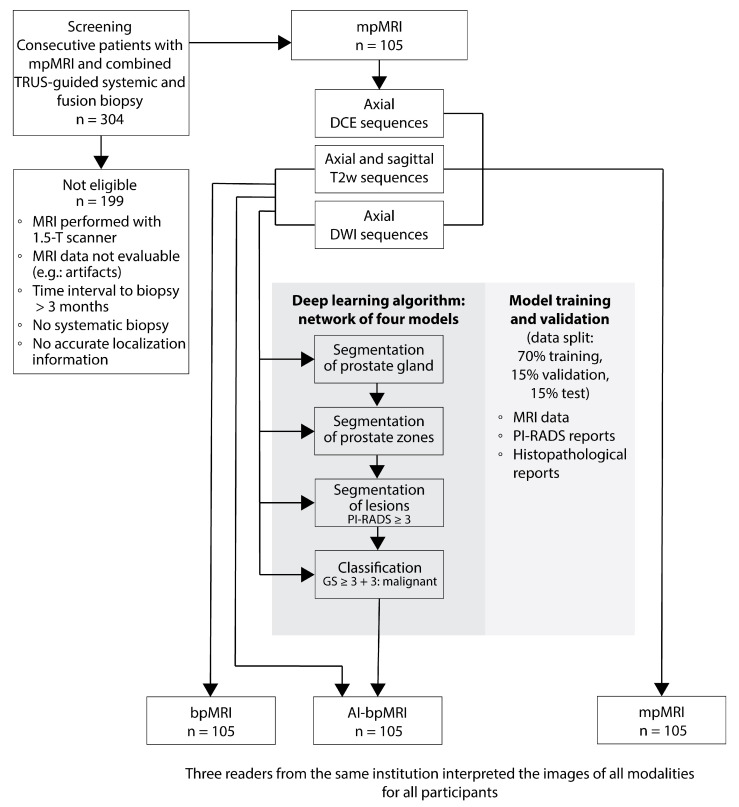
Flowchart of the retrospective multireader multicase study design. AI-bpMRI = artificial intelligence-assisted biparametric MRI, bpMRI = biparametric MRI, DCE = dynamic contrast-enhanced imaging, DWI = diffusion-weighted imaging, GS = Gleason score, MRI = magnetic resonance imaging, PCa = prostate cancer, PI-RADS = Prostate Imaging Reporting and Data System, T2 = T2-weighted imaging, TRUS = transrectal ultrasound.

We retrospectively included men who underwent mpMRI with following combined TRUS guided 12-core systematic and targeted fusion-guided biopsy at our institution. Exclusion criteria are listed in Figure 1. Consecutive series of cases and controls were enrolled according to the true disease status as determined by histopathologic examination (on average, 19 ± 13 days after mpMRI), including 52 men (50%) without PCa (healthy controls) and 53 men (50%) with Gleason score (GS) ≥ 3 + 3 PCa.

### 2.2. MRI Protocol

Multiparametric MRI of the prostate was performed with a 3-T MRI scanner (MAGNETOM Skyra and MAGNETOM Vida fit, Siemens Healthineers AG, Forchheim, Germany) using an 18-channel external surface coil. In the absence of contraindications, hyoscine butylbromide (Buscopan^TM^, Boehringer Ingelheim Pharma GmbH, Ingelheim, Germany) was given intravenously at a weight-dependent dose of 20–40 mg to reduce bowel and smooth muscle movements. The scan geometry was according to the recommendations of PI-RADS V2.1 with a slice thickness of 3 mm and no slice gap. The acquired DWI b-values were 50, 500, and 800 s/mm^2^ and the calculated b-value was 2000 s/mm^2^. For DCE, men received 0.1 mmol/kg gadolinium-based contrast agent (Gadobutrol, Bayer, Leverkusen, Germany) at 2.5 mL/s with a temporal resolution of 5 s.

### 2.3. AI Decision

We trained a deep learning-based algorithm, consisting of a sequential network of four distinct models, to detect malignant prostate lesions (Prostate.Carcinoma.ai 0.1.0©, FUSE-AI GmbH, Hamburg, Germany). Three networks with an anisotropic U-net architecture (aniso-3DUNET) [16] were used to segment (a) the prostate gland, (b) the peripheral and transitional zone, and (c) suspicious lesions. The fourth model, with a Visual Geometry Group (VGG) net-based three-dimensional convolutional neural network (3D-CNN) architecture [17,18], was used for lesion classification. Image segmentation and classification were performed using the open-source software TensorFlow (version 2.10.0, modules “tf.” and “tf.keras”, Google Ireland Limited, Dublin Ireland) and the python package batchgenerators (German cancer research center, Heidelberg, Germany) [19]. The network predictions were overlaid on the axial T2-weighted (T2w) images, creating segmentations with different colors to highlight likely malignant and benign prostate regions. Readers provided their interpretation after viewing the AI results (Figure 1).

### 2.4. Network Training and Evaluation

A total of 712 patient records were annotated (different from the study patients). The data included de-identified private and public data sources, including PROMISE12 [20], PROSTATEx/PROSTATEx-2 [21], and the Medical Segmentation Decathlon dataset [22]. All sources included segmentation of the prostate gland, while only a subset included annotations for zones, lesions (PI-RADS ≥ 3), and histopathological findings from biopsy or prostatectomy (ground truth). Lesions with a GS of 3 + 3 and higher were classified as malignant. Cases with inconclusive findings were excluded. The models were trained in the following way. The data were split into 70% for training, 15% for validation, and 15% for testing (a, b, c, d). Binary cross entropy (BCE) (a), 0.25 BCE + 0.75 (1-Dice) (b), 0.5 BCE + 0.5 (1-Dice) (c), and categorical cross entropy (CCE) were used as loss functions. Training was conducted with Adam optimizer and a fixed learning rate of 8 × 10^−5^ (a, b, c) and a scheduled learning rate with start 1 × 10^−5^, decay rate 0.96, decay steps 1 × 10^5^, staircase = true (d). Batch sizes were 2 (a, b, c) and 10 (d) and the numbers of epochs were 300 (a), 150 (b), 121 (c), and 100 (d). The AI-segmentation algorithms achieved a voxel-based Dice coefficient of 0.85 for the prostate gland, 0.74 for the prostate zones, and 0.25 for lesions. They classified malignant lesions with a sensitivity of 89% and a specificity of 81%. In the study cohort, AI alone classified GS 3 + 4 PCa lesions with a sensitivity of 97% (34/35) and a specificity of 23% (16/70).

### 2.5. Radiological Assessment

**Figure 2:**
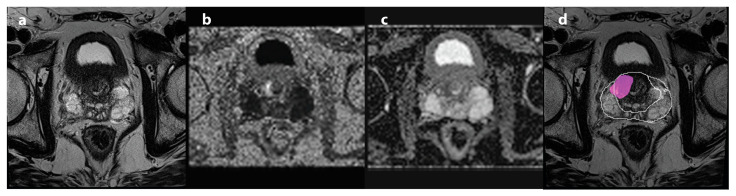
MRI examination obtained from a 70-year-old patient with an elevated PSA of 7.4 ng/mL. Image of the axial T2w sequence (**a**), the axial calculated b = 2000 s/mm^2^ DWI sequence (**b**), the axial ADC map (**c**), and the axial T2w sequences with the AI-generated map showing prostate and lesion segmentation (**d**). The combined 12-core TRUS guided fusion biopsy proved GS 3 + 4 prostate cancer only in targeted biopsy core of suspicious lesion. ADC = apparent diffusion coefficient, DWI = diffusion-weighted imaging, GS = Gleason score, TRUS = transrectal ultrasound.

Readers were provided with (a) bpMRI scans of axial and sagittal T2w sequences combined with axial DWI sequences; (b) mpMRI scans of axial and sagittal T2w sequences combined with axial DWI and DCE sequences; and (c) AI-bpMRI scans of axial and sagittal T2w sequences combined with axial DWI sequences and an AI-generated map showing prostate and lesion segmentation (Figure 2).

To compare the diagnostic accuracy of the three MRI modalities, we used a factorial multireader multicase (MRMC) study design [23,24]. Three radiologists with low (R_low_: >50 mpMRI prostate examinations), medium (R_med_: >200 mpMRI prostate examinations), and high (R_high_: >3000 mpMRI prostate examinations) experience independently interpreted the images of all participants in blinded reading sessions without access to clinical information. Both R_low_ and R_med_ were trained as part of the educational programme in our institute, with approximately 50 mpMRI prostate examinations prior to this study.

All readers viewed images in random case order with at least a four-week interval between each MRI modality. The disease status of 12 prostate zones was rated according to the PI-RADS-2.1 classification [4,25]. These zones included the peripheral and transitional zones located at the left and right sides of the base, mid-portion, and apex of the prostate gland. The overall PI-RADS score was then determined for each participant based on these ratings. Images were evaluated and findings documented using mint Lesion^TM^ (Mint Medical, version 3.8.0, Heidelberg, Germany).

### 2.6. Reference Standard

Diagnostic accuracy was evaluated by assessing the true disease status of PCa based on histopathological findings from biopsy. Prostate tissue samples for histopathological examination were systematically obtained from all included men with suspected PCa according to a clinical routine via transrectal ultrasound-guided 12-core biopsy from the prostate zones. In addition, targeted biopsy samples based on the previous mpMRI findings were taken. All biopsies were processed following routine procedures for formalin-fixed and paraffin-embedded (FFPE) tissues. Haematoxilin and eosin (H&E)-stained sections of FFPE tissues were evaluated with light microscopy. The overall participant-level GS was determined based on all individual scores [26,27]. Only biopsy results from cores taken from the same or adjacent prostate zone as that considered for the PI-RADS score were used as reference for correlation. We used this approach to avoid misclassification of cases, in which e.g., for PCa positive cores were in opposite regions located as the PI-RADS ≥ 3 lesions. Alternative diagnoses in those without PCa were benign prostate hyperplasia, prostatitis, and precancerous lesions.

### 2.7. Study End Points

The primary study objective was to determine the difference in readers’ mean participant-level area under the receiver operating characteristic (ROC) curve (AUC) between AI-bpMRI, mpMRI, and bpMRI. The main secondary study objective was to test the non-inferiority of the readers’ mean participant-level AUC with AI-bpMRI versus mpMRI. Additional secondary objectives were sensitivity, specificity, positive and negative likelihood ratios, and partial AUCs (pAUCs) at a sensitivity level of >80%. Binary accuracy metrics were applied to a PI-RADS-2.1 classification threshold of ≥3 for a positive test result. To ensure a comprehensive evaluation of disease manifestations, we evaluated all outcomes for both GS ≥ 3 + 4 and GS ≥ 3 + 3 PCa.

### 2.8. Statistical Analysis

The primary null hypothesis was that there would be no difference between readers’ mean AUCs with AI-bpMRI, mpMRI, or bpMRI. A sample of 3 readers and 98 cases (49 men with and 49 without the presence of PCa) was calculated to be adequate to provide 80% power with a type I error rate of 5%, to detect a minimum difference in readers’ mean AUC of 20%, assuming moderate inter- and intra-reader variability of 5% and 2.5%, respectively, a moderate correlation between readers of 0.47, and an expected mean readers’ AUC of 0.75. Sample size was calculated using the Obuchewski–Rockette sample size estimation [28] with the MRMCsamplesize R package version 1.0.0 [29]. The null hypothesis of the main secondary study objective of non-inferiority of AI-bpMRI to mpMRI was that the mean AUC with AI-bpMRI would be inferior to that with mpMRI. Non-inferiority of AI-bpMRI was established if the lower limit of the 95% confidence interval (CI) of the difference between readers’ mean AUC_AI-bpMRI_ and AUC_mpMRI_ was greater than −0.05 (H_A_: AUC_AI-bpMRI_–AUC_mpMRI_ > −0.05) [30]. Based on the assumption that in a retrospective study design, sensitivity can be properly determined with at least 50 participants with, and 50 without the disease [24], a total sample size of 105 participants was considered adequate. To account for variability between participants and readers and for correlation between readers, we analyzed the data using the MRMC-specific software MRMCaov R package version 0.3.0 [31,32], implementing the ANOVA approach of Obuchowki and Rockette and the models of Hillis and colleagues [28,33]. We conducted an analysis of available cases. Readers were designated as fixed, and participants as random. Covariances were calculated using the jackknife method. Partial AUCs were calculated and compared using the R package pROC version 1.18.5 [34]. To correct for multiple testing on reader experience-specific AUCs, the significance level was adjusted to *p* < 0.017 using the Bonferroni procedure.

## 3. Results

We evaluated 105 men (average age 66 ± 7 years) with elevated prostate-specific antigen (average prostate-specific antigen [PSA] 9.4 ± 6.8 ng/mL) and/or abnormal prostate findings on digital rectal examination, who underwent mpMRI and histopathological examination (on average, 19 ± 13 days after mpMRI) (Table 1).

### 3.1. Area Under the ROC Curve

**Figure 3:**
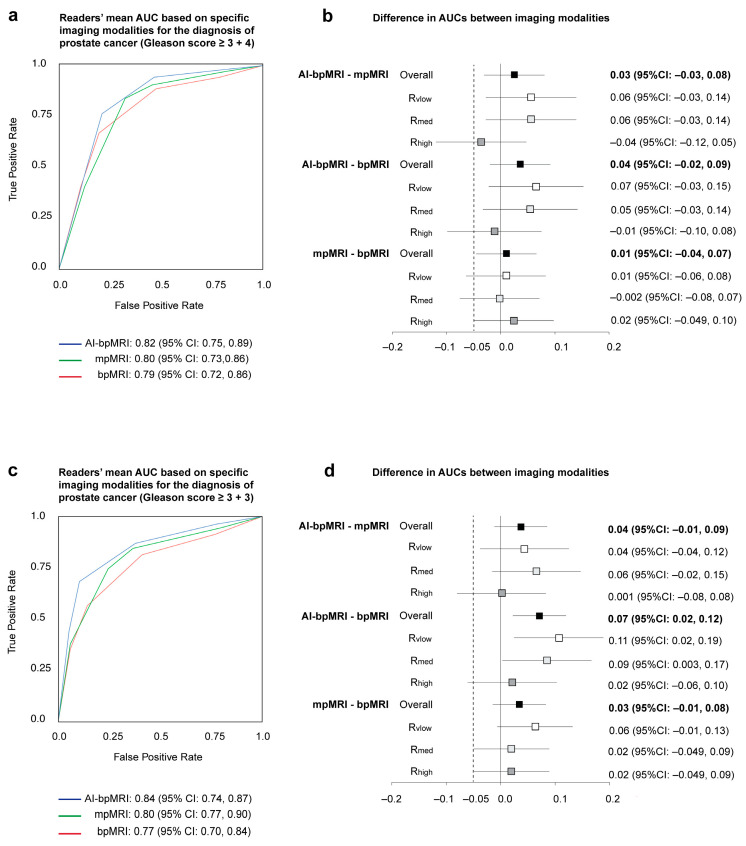
Diagnostic performance of three different MRI modalities for the detection of (**a**,**b**) Gleason score ≥ 3 + 4 and (**c**,**d**) Gleason score ≥ 3 + 3 PCa. (**a**,**c**) Patient-level areas under receiver operating characteristic (ROC) curves (AUCs) based on modality-specific assessments by three readers of 105 cases using multireader multicase analysis. (**b**,**d**) Overall and reader-specific pairwise comparison of ROC AUCs from three different MRI modalities assessed by multireader multicase analysis (fixed readers, random cases). The non-inferiority margin of −0.05 is marked by a dashed line. AI-bpMRI = artificial intelligence-assisted biparametric MRI, bpMRI = biparametric MRI, MRI = magnetic resonance imaging, mpMRI = multiparametric MRI, R_high_ = high experience reader, R_vlow_ = very low experience reader, R_med_ = medium experience reader.

Overall, AI-bpMRI detected GS ≥ 3 + 4 PCa without significantly higher accuracy than mpMRI and bpMRI (difference in mean AUCs: 0.03 [95% CI: −0.03, 0.08], *p* = 0.37 and 0.04 [95% CI: −0.02, 0.09], *p* = 0.20, respectively). Non-inferiority of AI-bpMRI to mpMRI was demonstrated via the overall AUC for the detection of both GS ≥ 3 + 4 and GS ≥ 3 + 3 PCa. The difference in mean AUCs between mpMRI and bpMRI was 0.01 [95% CI: -0.04, 0.07], *p* = 0.70 for the detection of GS ≥ 3 + 4 PCa and 0.03 [95% CI: -0.01, 0.08], *p* = 0.17 for the detection of GS ≥ 3 + 3 PCa. In addition, with the use of AI-bpMRI, GS ≥ 3 + 3 PCa was detected with a significantly higher accuracy than with bpMRI (mean AUC: 0.84 vs. 0.77; difference: 0.07 [95% CI: 0.02, 0.12], *p* = 0.004) (Figure 3).

**Figure 4:**
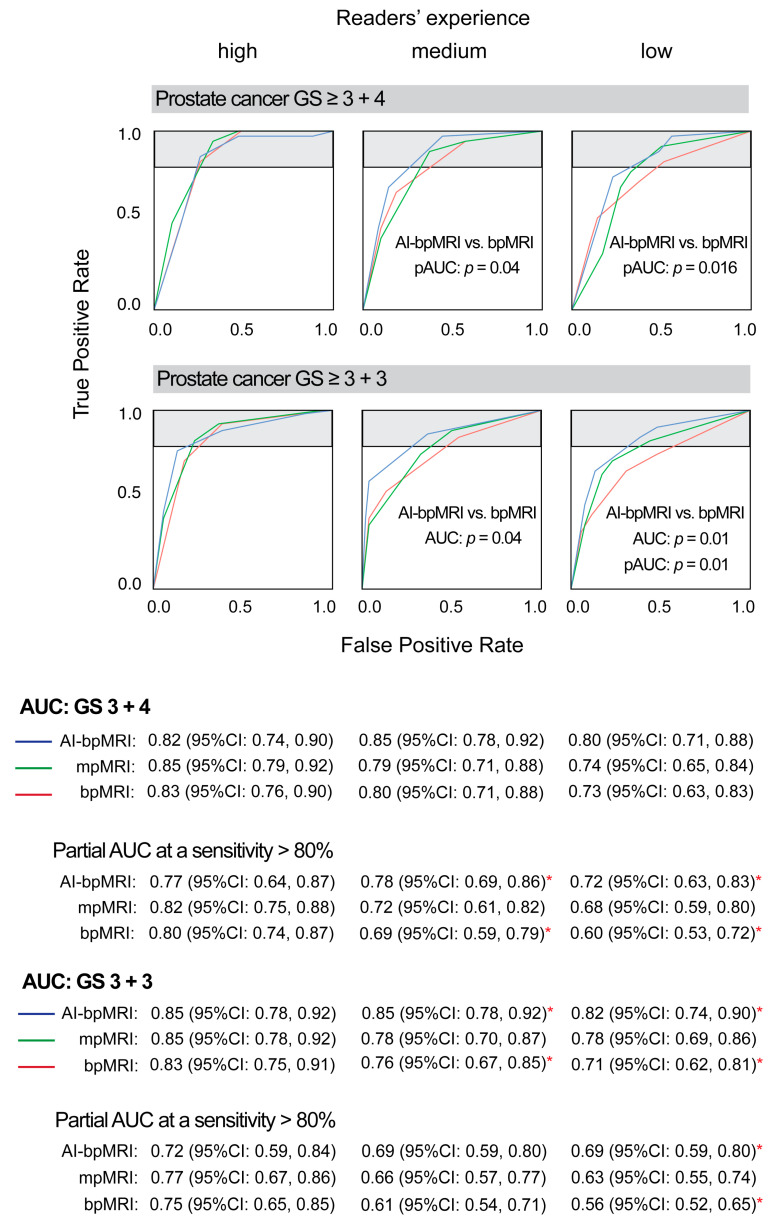
Diagnostic accuracy expressed in receiver operating characteristic curves of three different MRI modalities specified for three readers with very low, medium, and high experience, respectively. Areas of high sensitivity (>80%) are highlighted in grey. Differences in AUCs/pAUCs with *p* < 0.05 between MRI modalities are marked with red asterisks. AUC = area under receiver operating characteristic (ROC) curve, AI-bpMRI = artificial intelligence-assisted biparametric MRI, bpMRI = biparametric MRI, GS = Gleason score, MRI = magnetic resonance imaging, mpMRI = multiparametric MRI, pAUC = partial area under ROC curve.

Regarding experience specific AUCs, the advantage of AI-bpMRI over mpMRI and bpMRI was highest in low and medium experienced readers. In the highly experienced reader, there was almost no difference between the AUCs of AI-bpMRI and bpMRI or mpMRI, and non-inferiority of AI-bpMRI to mpMRI was not observed (Figure 3b,d and Figure 4).

In terms of pAUC, which covers the range of high sensitivity (>80%), there was an advantage in the pAUC of AI-bpMRI over bpMRI for the detection of GS 3 + 4 PCa in both medium and low experienced readers. However, only the benefit for the less experienced readers reached significance (pAUC: 0.78 vs. 0.69, *p* = 0.04 and 0.72 vs. 0.60, *p* = 0.016, respectively). The low experienced reader detected GS ≥ 3 + 3 PCa with a significantly higher accuracy by using AI-bpMRI than with bpMRI (pAUC: 0.69 vs. 0.56, *p* = 0.01) (Figure 4).

### 3.2. Binary Accuracy Measures

Binary classification performance in the detection of GS ≥ 3 + 4 PCa did not differ between MRI modalities. However, GS ≥ 3 + 4 PCa was detected with higher sensitivity but lower specificity than GS ≥ 3 + 3 across all MRI modalities. GS ≥ 3 + 3 PCa was detected with an overall sensitivity of 87% with AI-bpMRI, 84% with mpMRI, and 81% with bpMRI. The difference in sensitivity between AI-bpMRI and bpMRI was 5.7% (95% CI: 0.3%, 11.0%) with *p* = 0.04, above the threshold of 0.017. There were also no significant differences between MRI modalities in terms of specificity and likelihood ratios (Table 2).

**Figure 5:**
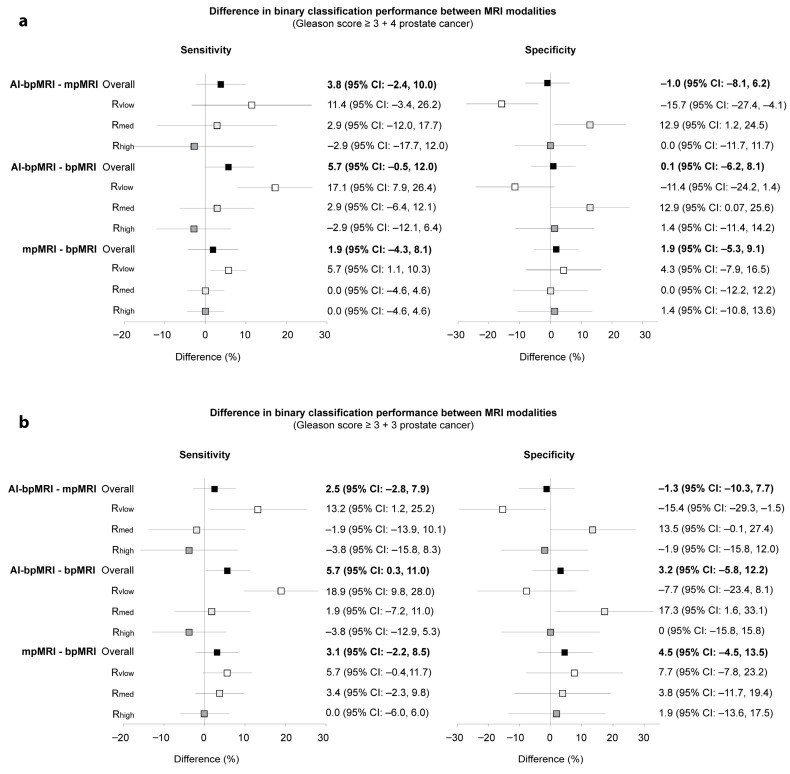
Comparison of the binary classification (PI-RADS 1, 2 vs. 3–5) performance of different MRI modalities for the diagnosis of PCa of (**a**) GS ≥ 3 + 4 and (**b**) GS ≥ 3 + 3. Forest plots show the differences in sensitivity and specificity between the MRI modalities as a summary result from the multireader multicase analysis (3 readers, 105 participants) and for each of the readers with very low, medium, and high experience, respectively. AI-bpMRI = artificial intelligence-assisted biparametric MRI, bpMRI = biparametric MRI, GS = Gleason score, MRI = magnetic resonance imaging, mpMRI = multiparametric MRI, R_high_ = high experience reader, R_vlow_ = very low experience reader, R_med,_ = medium experience reader.

The sensitivity of the low experienced reader in the detection of GS ≥ 3 + 4 PCa was increased with AI-bpMRI compared to mpMRI, but at the expense of specificity (sensitivity: 11.4% [*p* = 0.13], specificity: −15.7% [*p* = 0.008]). In the medium experienced reader, AI-bpMRI increased specificity compared to mpMRI (12.9%, *p* = 0.03). Similar results were found for AI-bpMRI compared to bpMRI (increase in sensitivity for low experienced reader: 17.1% [*p* < 0.001] with a decrease in specificity (−11.4% [*p* = 0.08]) and an increase in specificity for medium experienced reader (12.9% [*p* = 0.049]). For the highly experienced reader, there were no significant differences in sensitivity and specificity between the three MRI modalities. Similar results for reader experience specific differences in sensitivity and specificity were found for the detection of GS ≥ 3 + 3 PCa (Figure 5).

## 4. Discussion

We compared the diagnostic accuracy of AI-bpMRI with the current state-of-the art methods of bpMRI and mpMRI for the detection of PCa, using an MRMC study design. We considered different grades of PCa and different levels of reader experience. AI-assisted bpMRI was non-inferior to mpMRI for detection of both GS ≥ 3 + 4 and GS ≥ 3 + 3 PCa and showed a significant advantage over bpMRI in detecting GS ≥ 3 + 3 PCa. The accuracy of the highly experienced reader was almost unaffected by AI assistance. For the less experienced reader, the increase in sensitivity with AI-bpMRI was at the expense of specificity; for the moderately experienced reader, AI-bpMRI improved overall diagnostic performance by increasing specificity without affecting sensitivity. This is consistent with the results of recently published studies, which also observed improvement in diagnostic performance through the use of AI, although the effect of different levels of experience was not specifically investigated [11,35]. Omission of the DCE-MRI sequence offers the advantages of shorter examination times, no contrast-related operational issues such as renal function and allergy assessment, and no contrast-related side effects [36]. However, DCE can serve as “backup” to detect smaller cancers, especially in the peripheral zone, when DWI is degraded [4,30,36]. When DWI or T2-weighted sequences are of suboptimal quality, the diagnostic value of AI-bpMRI is markedly reduced. In such situations, a high-quality dynamic contrast-enhanced (DCE) sequence can improve the PI-QUAL v2.0 score to an acceptable image quality level (PI-QUAL score 2). This substantially reduces the rate of repeat examinations [37].

In addition, a previous study reported the potential for missing PCa lesions on bpMRI in patients with prostatitis or previous prostate biopsy [30]. However, evidence of the dispensability of DCE is supported by our finding no difference between bpMRI and mpMRI in terms of diagnostic accuracy for detecting GS ≥ 3 + 4 PCa. Nevertheless, with decreasing reader experience, there was a slightly increasing advantage of mpMRI over bpMRI in terms of sensitivity and specificity. This is consistent with the literature, where less experienced readers required DCE to increase sensitivity and AUC [38]. AI assistance more than compensated for the disadvantage of not using DCE in the moderately experienced reader and significantly increased the sensitivity in the less experienced reader. However, this should not lead to the conclusion that AI can replace reader training or compensate for poor image quality. AI-supported diagnosis of PCa by low experienced readers may serve as valuable training but should not be used to guide treatment decisions and thus increase the risk of overdiagnosis. Finally, our study shows that AI assistance is not necessary for advanced readers when image acquisition parameters and image quality comply with PI-RADS V2.1 recommendations. A previous study showed that the performance of deep learning networks increases with the size of the training datasets [39]. It is therefore reasonable to expect that AI assistance will continue to improve as training datasets grow. The goal of AI development should be to reliably help moderately experienced readers reach the level of highly experienced readers. Although transparency of AI networks should be ensured as far as possible to encourage implementation in clinical practice, full traceability at the user level will be impossible to achieve. Even with AI assisted reporting, clinical parameters such as PSA density should continue to be considered alongside the results from AI-bpMRI or mpMRI when deciding whether a biopsy of detected lesions is warranted [40].

It is essential for users to acknowledge the intention of the used algorithm. This AI was trained to achieve high sensitivity while allowing lower target values for specificity. This decision accounts for the relatively low DICE coefficient observed for lesion segmentation. Our primary aim was to ensure the detection of all significant prostate cancers, including borderline and equivocal cases. However, such high sensitivity almost inevitably leads to a relatively large number of false-positive lesions. As the AI was designed to assist radiologists, it remains their responsibility to identify and exclude false positives and thereby maintain adequate specificity and avoid an increase in biopsy rates.

Our study has some limitations. First, although the readers had a wide range of experience, they cannot be considered as representative of the general reader population because they were from a single institution and numbered only three readers. Therefore, the readers were designated as fixed factors for the MRMC analysis. However, these study results cannot be generalized. Second, a selection bias was present in our study population, since patients underwent combined biopsy after considering results of the Rotterdam Prostate Cancer Risk Calculator. It is essential to be aware of this selection bias when comparing our results with those of other studies. It would not be appropriate to extrapolate our findings to a screening population with a considerably lower incidence of prostate cancer.

## 5. Conclusions

In conclusion, replacing contrast enhancement with AI assistance at bpMRI was non-inferior to mpMRI in detection prostate cancer. The effect of AI assistance differed considerably according to reader experience. Future research involving more readers and more data from different institutions is needed to generalize the results to an appropriate reader population. Nevertheless, our results provide preliminary insights into areas in which AI-bpMRI could be most effectively applied in future. Based on our results, the benefits of AI-bpMRI were most evident for radiologists with low to medium experience. Therefore, implementing AI-bpMRI could substantially improve patient care in regions where expertise in prostate MRI interpretation is limited. Furthermore, our findings suggest that contrast material can safely be omitted for the sole purpose of detecting suspicious prostate cancer areas. This would, in turn, support the use of AI-bpMRI as a triage tool prior to mpMRT in high-volume centers.

## Figures and Tables

**Table 1:** Participant demographics and clinical characteristics.

Characteristic (N = 105)	
Age (y) *	66 ± 7
PSA level (ng/mL) *	9.4 ± 6.8
Suspiciously elevated PSA level	38 (36)
Abnormal rise in PSA	59 (56)
Suspicious digital rectal examination	3 (3)
Staging following positive biopsy	2 (2)
Active surveillance	3 (3)
Histopathology	
No PCa	52 (50)
GS 3 + 3	18 (17)
GS 3 + 4	21 (20)
GS 4 + 3	3 (3)
GS 4 + 4	10 (10)
GS 4 + 5	1 (1)

Unless otherwise specified, data are numbers of participants with percentages in parenthesis. GS = Gleason score, PCa = prostate carcinoma, PSA = prostate-specific antigen. * Data are means ± standard deviations.

**Table 2:** Binary classification performance of different MRI modalities for the diagnosis of prostate cancer.

	AI-bpMRI	mpMRI	bpMRI
**Gleason score** ≥ **3 + 4**			
Sensitivity, %	94.3 (88.0, 100)	90.5 (85.1, 95.9)	88.6 (82.6, 94.5)
Specificity, %	53.3 (44.0, 62.7)	54.3 (45.4, 63.2)	52.4 (43.5, 61.3)
LR+	2.0 (1.6, 2.7)	2.0 (1.6, 2.6)	1.9 (1.5, 2.4)
LR−	0.1 (0.0, 0.3)	0.2 (0.1, 0.3)	0.2 (0.1, 0.4)
**Gleason score** ≥ **3 + 3**			
Sensitivity, %	86.8 (78.9, 94.7) *	84.3 (77.3, 91.2)	81.1 (73.2, 89.1) *
Specificity, %	62.2 (52.3, 72.0)	63.5 (54.1, 72.8)	59.0 (49.5, 68.4)
LR+	2.3 (1.7, 3.2)	2.3 (1.7, 3.4)	2.0 (1.5, 3.0)
LR−	0.2 (0.2, 0.4)	0.2 (0.1, 0.4)	0.3 (0.1, 0.5)

Data are expressed as overall percentages or ratios with 95% confidence intervals in brackets. MRI images from three different modalities of 105 men were graded by three readers with very low, medium, and high experience, respectively. Analysis was performed using a multireader, multicase analysis (fixed readers, random cases). Accuracy metrics apply to a PI-RADS-2.1 classification threshold of ≥3 for a positive test result. * *p* value for the difference < 0.05. LR+ = likelihood ratio for a positive test result, LR− = likelihood ratio for a negative test result, AI-bpMRI = artificial intelligence-assisted biparametric MRI, bpMRI = biparametric MRI, MRI = magnetic resonance imaging, mpMRI = multiparametric MRI.

## Data Availability

The raw data supporting the conclusions of this article will be made available by the authors on request.

## Abbreviations

The following abbreviations are used in this manuscript:

AI-bpMRI	AI-assisted biparametric MRI
PCa	prostate cancer
mpMRI	multiparametric MRI
bpMRI	biparametric MRI
AUC	area under the receiver operating characteristic curves
pAUCs	partial AUCs
ROC	receiver operating characteristic
GS	Gleason score
T2w	T2-weighted
DWI	diffusion-weighted imaging
DCE	dynamic contrast-enhanced imaging
PI-RADS	Prostate Imaging Reporting and Data System
VGG	Visual Geometry Group
BCE	binary cross entropy
CCE	categorical cross entropy
MRMC	multireader multicase
FFPE	formalin-fixed and paraffin-embedded
H&E	haematoxilin and eosin
CI	confidence interval
LR+	likelihood ratio for a positive test result
LR−	likelihood ratio for a negative test result
